# Abutting Left Atrial Appendage and Left Superior Pulmonary Vein Predicts Recurrence of Atrial Fibrillation After Point-by-Point Pulmonary Vein Isolation

**DOI:** 10.3389/fcvm.2022.708298

**Published:** 2022-02-15

**Authors:** Nándor Szegedi, Judit Simon, Bálint Szilveszter, Zoltán Salló, Szilvia Herczeg, Lili Száraz, Márton Kolossváry, Gábor Orbán, Gábor Széplaki, Klaudia Vivien Nagy, Mohammed El Mahdiui, Jeff M. Smit, Victoria Delgado, Jeroen J. Bax, Pál Maurovich-Horvat, Béla Merkely, László Gellér

**Affiliations:** ^1^Heart and Vascular Center, Semmelweis University, Budapest, Hungary; ^2^MTA-SE Cardiovascular Imaging Research Group, Heart and Vascular Center, Semmelweis University, Budapest, Hungary; ^3^Heart and Vascular Centre, Mater Private Hospital, Dublin, Ireland; ^4^Department of Cardiology, Leiden University Medical Center, Leiden, Netherlands; ^5^Department of Radiology, Medical Imaging Centre, Semmelweis University, Budapest, Hungary

**Keywords:** abutting, left atrial appendage (LAA), left superior pulmonary vein (LSPV), atrial fibrillation (AF), ablation electrophysiology, pulmonary vein isolation (PVI), success rate- catheter ablation, recurrence

## Abstract

**Introduction:**

The role of the spatial relationship between the left superior pulmonary vein (LSPV) and left atrial appendage (LAA) is unknown. We sought to evaluate whether an abutting LAA and LSPV play a role in AF recurrence after catheter ablation for paroxysmal AF.

**Methods:**

Consecutive patients, who underwent initial point-by-point radiofrequency catheter ablation for paroxysmal AF at the Heart and Vascular Center of Semmelweis University, Budapest, Hungary, between January of 2014 and December of 2017, were enrolled in the study. All patients underwent pre-procedural cardiac CT to assess left atrial (LA) and pulmonary vein (PV) anatomy. Abutting LAA-LSPV was defined as cases when the minimum distance between the LSPV and LAA was less than 2 mm.

**Results:**

We included 428 patients (60.7 ± 10.8 years, 35.5% female) in the analysis. AF recurrence rate was 33.4%, with a median recurrence-free time of 21.2 (8.8–43.0) months. In the univariable analysis, female sex (HR = 1.45; 95%CI = 1.04–2.01; *p* = 0.028), LAA flow velocity (HR = 1.01; 95%CI = 1.00–1.02; *p* = 0.022), LAA orifice area (HR = 1.00; 95%CI = 1.00–1.00; *p* = 0.028) and abutting LAA-LSPV (HR = 1.53; 95%CI = 1.09–2.14; *p* = 0.013) were associated with AF recurrence. In the multivariable analysis, abutting LAA-LSPV (adjusted HR = 1.55; 95%CI = 1.04–2.31; *p* = 0.030) was the only independent predictor of AF recurrence.

**Conclusion:**

Abutting LAA-LSPV predisposes patients to have a higher chance for arrhythmia recurrence after catheter ablation for paroxysmal AF.

## Introduction

Atrial fibrillation (AF) is the most common sustained cardiac arrhythmia, which leads to increased morbidity and mortality (1, 2). Pulmonary vein isolation (PVI) is the most effective treatment for drug-resistant symptomatic paroxysmal AF (3, 4). However, the success rate of catheter ablation varies considerably (5, 6). Optimal patient selection is of high importance in light of considerable AF recurrence and potential procedural complications (7). Numerous predictors of AF recurrence have been described, such as hypertension, left atrial (LA) enlargement, particular pulmonary vein (PV) anatomical variations, and certain left atrial appendage (LAA) morphologies (8–12). The role of both the LAA and PV anatomical variations in the efficacy of PVI procedures is verified by previous studies. However, the role of the anatomical relationship between these two neighboring structures [e.g. left superior pulmonary vein (LSPV) and LAA] is unknown. We aimed to evaluate whether abutting LAA-LSPV could play a role in AF recurrence after catheter ablation for paroxysmal AF.

## Methods

### Study Population

Consecutive patients, who underwent point-by-point radiofrequency catheter ablation for paroxysmal AF at the Heart and Vascular Center of Semmelweis University, Budapest, Hungary, between January of 2014 and December of 2017, were retrospectively enrolled in the study. All included patients underwent pre-procedural cardiac computed tomography (CT) for complex assessment of LA anatomy. Demographic, anthropometric, and medical data of all patients were collected. Patients with non-paroxysmal AF, repeated ablation procedure, non-diagnostic image quality of left atrial CT and not point-by-point ablation technique were excluded from the current study (Flowchart of patient enrollment in Supplementary Material).

All patients agreed to pre-procedural imaging, ablation procedure and provided written consent to data retrieval and analysis. The study protocol was reviewed and approved by the institutional ethics committee and was in accordance with the declarations of Helsinki.

### Cardiac CT Imaging

Computed tomography angiography examinations were performed with a 256-slice scanner (Brilliance iCT 256, Philips Healthcare, Best, The Netherlands) with prospective ECG-triggered axial acquisition mode. For cardiac CT, 100–120 kV with 200–300 mAs tube current was used based on patient anthropometrics. Image acquisition was performed with 128 x 0.625 mm detector collimation and 270 ms gantry rotation time. For heart rate control, a maximum of 50–100 mg metoprolol was given orally or 5–20 mg intravenously, if necessary. Mid-diastolic triggering was applied with 3–5% padding (73–83% of the R-R interval) in all included patients. Iomeprol contrast material (Iomeron 400, Bracco Ltd, Milan, Italy) was used with an 85–95 ml contrast agent at a flow rate of 4.5–5.5 ml/s from antecubital vein access via 18-gauge catheter using a four-phasic protocol. Bolus tracking in the LA was used to obtain proper scan timing. Sublingual nitroglycerin (0.8 mg) was given between the native and CT examinations. CT data sets were reconstructed with 0.8 mm slice thickness with 0.4 mm increments.

After defining LA borders with caution to the orifices of the pulmonary veins and the level of the mitral valve, we measured total LA and LAA volumes, LAA orifice area and determined LAA morphologies based on three-dimensional volume-rendered images using a semiautomated software package (EP Planning, Philips IntelliSpace Portal, Philips Healthcare, Best, The Netherlands).

Since assessment of LAA morphology can be highly subjective, LAA morphologies were determined by consensus reading of three expert readers using rigorous definitions in order to minimize inter-observer variability. LAA morphologies were classified in four different types as previously described: (1) Cauliflower, if LAA has a limited length and the distal width exceed the proximal width; (2) Windsock, if the primary structure is one dominant lobe with sufficient length; (3) Chicken wing, if the dominant lobe has an obvious bend in the proximal or middle part; and (4) Swan if LAA has a second sharp curve folding the dominant lobe back (13).

**Figure 1:**
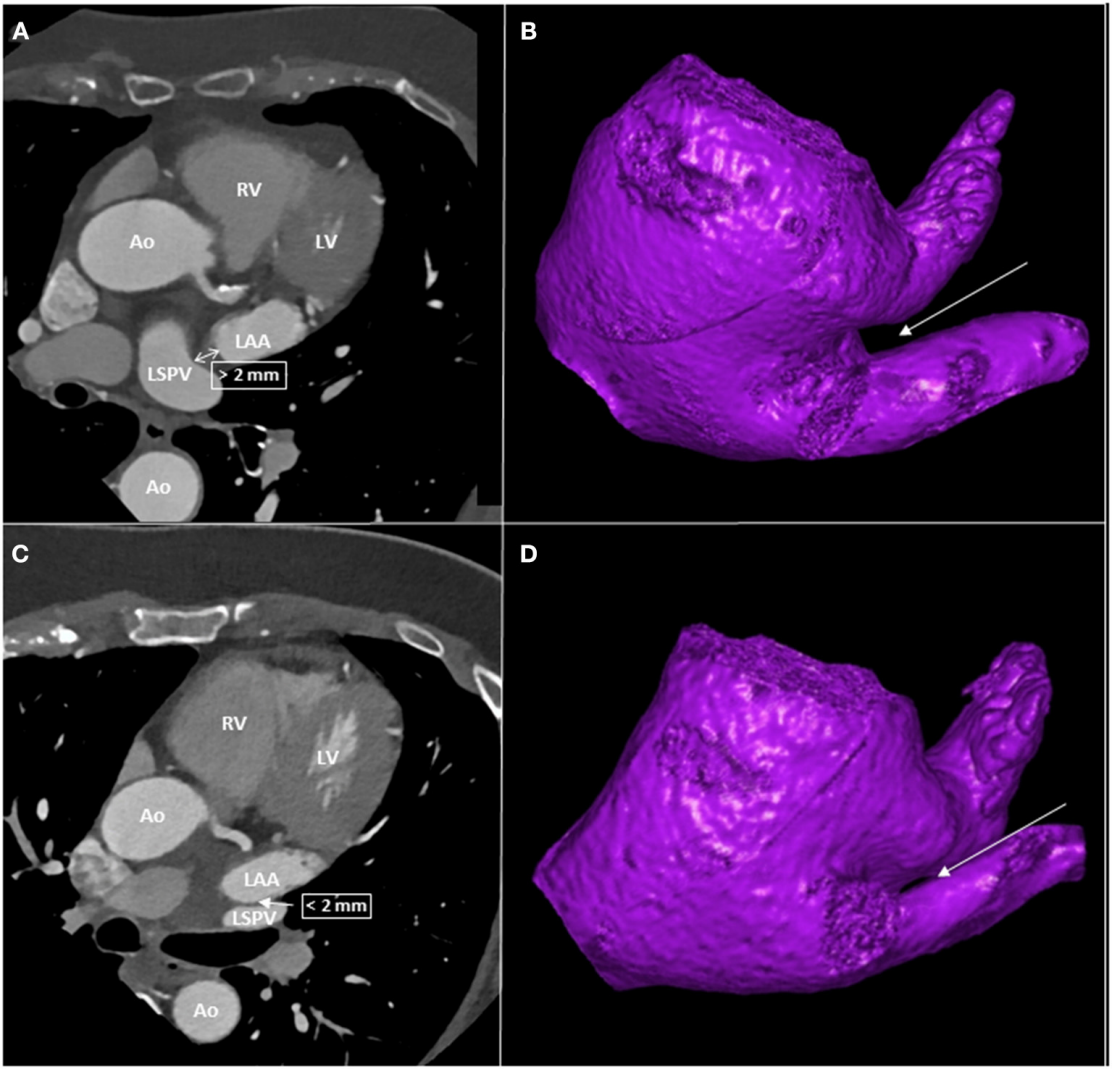
CT images of patients with and without abutting LAA-LSPV. **(A)** left atrial CT of a patient with non-abutting LAA-LSPV. **(B)** three-dimensional reconstruction of left atrium from caudal view, with a highlight on non-abutting LAA-LSPV. The LIPV was removed to give a better visualization of the LSPV. **(C)** left atrial CT of a patient with abutting LAA-LSPV. **(D)** three-dimensional reconstruction of left atrium from caudal view, with a highlight on abutting LAA-LSPV. The LIPV was removed to give a better visualization of LSPV. The arrows indicate the site of distance measurement between LAA and LSPV. Ao, aorta, CT, computed tomography, LAA, left atrial appendage, LV, left ventricle, LIPV, left inferior pulmonary vein, LSPV, left superior pulmonary vein, RV, right ventricle.

The spatial relationship between LAA and LSPV was determined using two-dimensional axial CT images. Abutting LAA-LSPV was defined as cases when the LSPV touched the posterior aspect of LAA, more precisely when the minimal distance between the endocardial borders of the two structures was less than 2 mm. Those cases where the distance between the posterior border of LAA and the anterior border of the LSPV was more than 2 mm were defined as non-abutting LAA-LSPV (Figure 1).

### Transesophageal Echocardiography

Maximum 24 h before ablation, all patients underwent Transesophageal echocardiography (TEE) examination to exclude the presence of LAA thrombus. iE33 and Epiq 7C (Philips Medical System, Andover, MA) systems equipped with S5-1 phased array and X7-2t matrix TEE transducers were used. TEE was performed during conscious sedation. The LAA was imaged from 0, 45, 90, and 135° views to detect spontaneous echo contrast, sludge or thrombus. Subsequently, a sample volume was placed at the middle portion of the LAA and the peak velocity of the LAA outflow was measured.

### Catheter Ablation Procedure

Indications for AF ablation procedures were according to the current guidelines (3). All ablations were performed by experienced operators (>100 AF ablations/year). Conscious sedation was carried out in all cases with intravenous fentanyl, midazolam, and propofol. The basic vital parameters of the patients were monitored in all cases with non-invasive blood pressure measurements every 10 min and continuous pulse oximetry. Femoral venous access was used for all procedures. Transseptal puncture was performed under fluoroscopy guidance and pressure monitoring. Whenever transseptal puncture was complex, intracardiac echocardiography was used for direct visualization of the interatrial septum. All ablations were performed with the use of an electroanatomical mapping system (either CARTO, Biosense Webster, Inc., Diamond Bar, CA, USA; or ENSITE, St. Jude Medical, Inc., MN, USA), and LA fast anatomical map was merged/fused with the cardiac CT images to guide point-by-point ablation (temperature-controlled mode, 43°C, 25–35 W, irrigated 4 mm tip catheter). Ablations were conducted with non-contact force-sensing catheters in 2014 and 2015, while contact force-sensing catheters were used in 2016 and 2017. Complete electrical isolation of all PVs from the LA was the goal of each procedure. The left lateral ridge was ablated from the PV side in all cases. No additional ablations were performed beyond the PVI. PV disconnection was carefully examined in all cases, as both entrance and exit block was verified. Exit block was confirmed by the presence of local PV capture but the absence of LA capture during pacing inside the vein. The entrance block was confirmed by the elimination of previously visible sharp PV potentials; moreover, LAA pacing was performed to verify the absence of local PV potentials, and the remaining blunt signal in the LSPV is LAA far-field. All patients without complications were discharged the day after the procedure.

### Follow-Up and Definition of Recurrence

After the post-procedure discharge, outpatient clinical follow-up visits were scheduled at 3, 6, 12 months, and at least once yearly after that. Whenever patients experienced symptoms of arrhythmia, additional visits were scheduled. Follow-up visits included clinical assessment of the patients and 24 h Holter ECG monitoring. Follow-up data were registered in a structured reporting platform (Axis, Neumann Medical Ltd., Budapest, Hungary). Recurrence of AF was defined as the occurrence of atrial tachyarrhythmia that lasts for more than 30 s, documented by ECG. AF recurrences during the blanking period (first 90 days following catheter ablation) were not considered.

### Statistical Analysis

Categorical variables are expressed as frequencies, and continuous values are expressed as means ± standard deviation (SD) if normally distributed and median and interquartile range (IQR) if not normally distributed. The normality of continuous parameters was tested with the Shapiro-Wilk test. Since the rate of missing values was <10%, simple imputation was applied for compensation with mean values for continuous and most frequent values for binary and categorical variables. Tests for significance were conducted using Mann-Whitney, Wilcoxon, or Kruskal-Wallis tests for continuous variables and Pearson's chi-square or Fisher's exact tests for categorical variables. Two observers independently performed all CT measurements and were blinded to patient outcome data. Fifty patients were randomly selected for inter-observer agreement and analyzed using the inter-class correlation coefficient (ICC). To identify parameters associated with AF recurrence after catheter ablation, uni- and multivariable Cox proportional hazard regression model was executed. A multivariable model was built to determine the independent predictors of AF recurrence. The multivariable analysis included age, sex, body mass index (BMI), hypertension, hyperlipidemia, diabetes, prior stroke, or transient ischemic attack (TIA), obstructive coronary artery disease (CAD), thyroid gland disease, decreased estimated glomerular filtration rate (eGFR), impaired left ventricular ejection fraction (LVEF), body surface area indexed LA volume (iLAV), E/A ratio, LAA volume, LAA flow velocity, LAA orifice area, LAA morphology, and abutting LAA-LSPV. The event-free survival rate was estimated using the Kaplan-Meier method, and the log-rank test was applied for the comparisons between the various groups. Cumulative event rates were calculated with event or censoring times measured from the date of ablation. Two-tailed *p*-values smaller than 0.05 were considered significant.

All statistical analyses were performed in the R environment (version 3.6.1). Cox proportional hazard regression analysis was done using the “survival” package (version 3.1-8). Kaplan-Meier curve analysis and log-rank test were performed using the “survminer” (version 0.4.6) and “coin” (version 1.3-1) packages, respectively.

## Results

### Patient and Procedural Characteristics, Reproducibility of CT Assessment

Altogether 428 patients were included in the current analysis. The mean age was 60.7 ± 10.8 years, and 35.5% of the patients were female. Anthropometric data, cardiovascular comorbidities, imaging and procedural parameters are reported in Table 1. The reproducibility of the CT assessment was evaluated. The inter-class correlation coefficient for inter-observer variability for iLAV measurement was 0.995 (95%CI = 0.988–0.999; *p* < 0.001), for LAA volume measurement 0.992 (95%CI = 0.985–0.996; *p* < 0.001) and for the assessment of abutting LAA-LSPV 0.984 (95%CI = 0.979–0.992; *p* < 0.001). Procedure time, LA dwelling time and fluoroscopy time were 93.4 ± 27.9, 61.2 ± 23.7, and 9.2 ± 6.8 min, respectively.

**Table 1:** Patient characteristics.

	**All patients** **(*n* = 428)**	**No AF recurrence** **(*n* = 285)**	**AF recurrence** **(*n* = 143)**	***p***
Age (years)	60.7 ± 10.8	60.0 ± 11.2	61.9 ± 9.9	0.108
**Female**, ***n*** **(%)**	**152 (35.5)**	**89 (31.2)**	**63 (44.1)**	**0.010**
BMI (kg/m^2^)	28.7 ± 4.5	28.5 ± 4.3	29.1 ± 5.0	0.492
Hypertension, *n* (%)	287 (67.1)	187 (65.6)	100 (69.9)	0.385
Hyperlipidemia, *n* (%)	96 (22.4)	64 (22.5)	32 (22.4)	1.00
Diabetes, *n* (%)	56 (13.1)	40 (14.0)	16 (11.2)	0.451
Stroke/TIA, *n* (%)	34 (7.9)	21 (7.4)	13 (9.1)	0.572
Obstructive CAD, *n* (%)	37 (8.6)	22 (7.7)	15 (10.5)	0.364
Thyroid gland disease, *n* (%)	48 (11.2)	30 (10.5)	18 (12.6)	0.520
eGFR (ml/min/1.73 m^2^)	75.6 ± 14.4	75.6 ± 14.5	75.6 ± 14.3	0.590
**LVEF <50%**, ***n*** **(%)**	**26 (6.1)**	**12 (4.2)**	**14 (10.0)**	**0.031**
iLAV (ml/m^2^)	50.6 ± 15.3	49.9 ± 14.7	52.0 ± 16.3	0.220
E/A ratio	1.17 ± 0.40	1.18 ± 0.41	1.14 ± 0.39	0.368
LAAV (ml)	7.2 ± 3.0	7.1 ± 3.0	7.4 ± 3.0	0.282
LAA flow velocity (cm/sec)	37.4 ± 15.5	36.8 ± 15.2	38.7 ± 16.1	0.156
**LAA orifice area (mm** ^**2**^ **)**	**380.6 ±130.0**	**370.8 ±132.3**	**400.2 ±123.5**	**0.023**
LAA morphology				
Cauliflower, *n* (%)	227 (53.0)	155 (54.4)	72 (50.3)	0.776
Windsock, *n* (%)	130 (30.4)	86 (30.2)	44 (30.8)	
Chicken wing, *n* (%)	48 (11.2)	30 (10.5)	18 (12.6)	
Swan, *n* (%)	23 (5.4)	14 (4.9)	9 (6.3)	
LAA abutting LSPV, *n* (%)	232 (54.2)	145 (50.9)	87 (60.8)	0.064
Procedure time (min)	93.4 ± 27.9	95.7 ± 29.5	88.9 ± 24.0	0.074
LA time (min)	61.2 ± 23.7	62.2 ± 21.9	58.9 ± 28.4	0.424
Fluoroscopy time (min)	9.2 ± 6.8	9.4 ± 7.8	8.9 ± 4.2	0.855

*AF, atrial fibrillation; BMI, body mass index; CAD, coronary artery disease; eGFR, estimated glomerular filtration rate; iLAV, body surface area-indexed left atrial volume; LA, left atrium; LAA, left atrial appendage; LAAV, left atrial appendage volume; LVEF, left ventricular ejection fraction; LSPV, left superior pulmonary vein; TIA, transient ischemic attack. The bold variables were statistically significant*.

### Recurrence of AF

Recurrence of AF was found in 33.4% of the patients with a median recurrence-free time of 21.2 (8.8–43.0) months. The proportion of female patients was higher in the AF recurrence group (rate of female patients with AF recurrence and without AF recurrence was 44.1 and 31.2%, respectively; *p* = 0.010). Left ventricular ejection fraction (EF) was lower in the AF recurrence group (proportion of reduced ejection fraction in the non-recurrence group and recurrence group was 4.2 vs. 10.0%, respectively; *p* = 0.031). Indexed LA volume was 52.0 ± 16.3 ml for patients with AF recurrence and 49.9 ± 14.7 ml for patients without AF recurrence (*p* = 0.220). The LAA orifice area was higher in patients with AF recurrence compared to patients without AF recurrence (400.2 ± 123.5 vs. 370.8 ± 132.3; *p* = 0.023).

We found an excellent one and 2-year success rate both in cases ablated with and without contact force-sensing catheters (86.4% vs. 83.2% at 1 year, *p* = 0.419, and 74.5% vs. 74.5% at 2 years, *p* = 0.697). Moreover, there was no difference in the 1-year and 2-year success rates of cases performed with CARTO and EnSite system (86.4% and 83.2%, *p* = 0.419 at 1 year; 74.5% and 74.5%, *p* = 0.894 at 2 years).

### Predictors of AF Recurrence

Cox proportional hazards regression analysis was performed to analyze the associations between the investigated parameters and AF recurrence.

In the univariable analysis, female sex (HR = 1.45; 95%CI = 1.04–2.01; *p* = 0.028), LAA flow velocity (HR = 1.01; 95%CI = 1.00–1.02; *p* = 0.022), LAA orifice area (HR = 1.00; 95%CI = 1.00–1.00; *p* = 0.028), and abutting LAA-LSPV (HR = 1.53; 95%CI = 1.09–2.14; *p* = 0.013) were associated with AF recurrence.

In the multivariable analysis, abutting LAA-LSPV (adjusted HR = 1.55; 95%CI = 1.04–2.31; *p* = 0.030) was the only independent predictor of AF recurrence.

**Figure 2:**
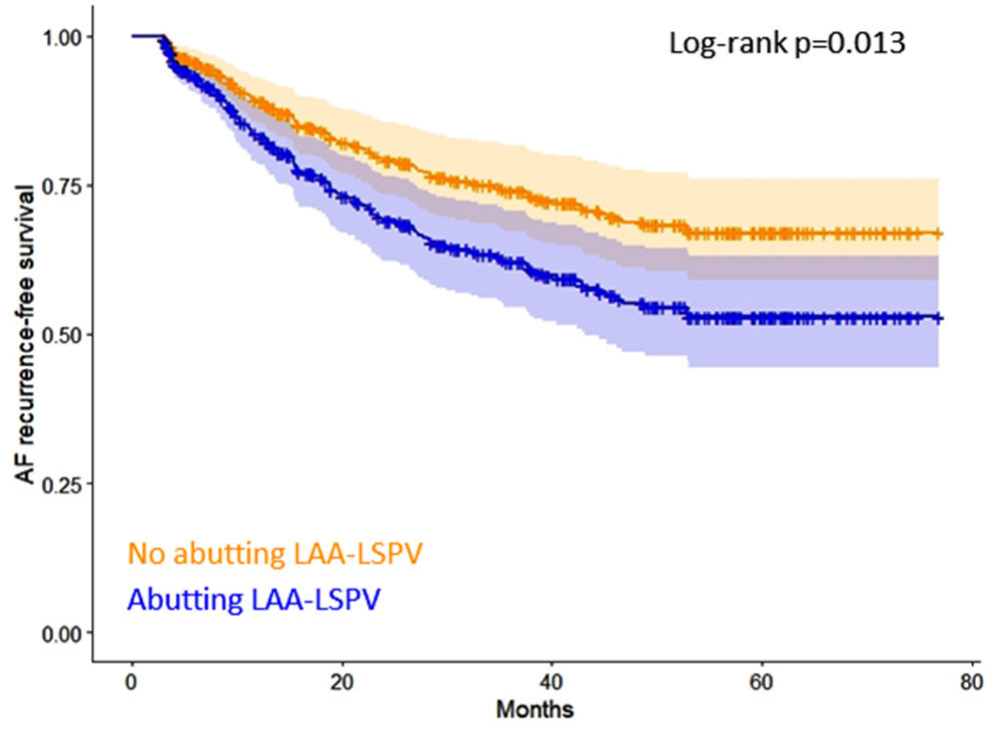
Kaplan-Meier curves of AF recurrence-free survival based on the presence or absence of abutting LAA-LSPV. AF, atrial fibrillation, LAA, left atrial appendage, LSPV, left superior pulmonary vein.

Kaplan-Meier curves of AF recurrence-free survival based on the presence or absence of abutting LAA-LSPV are shown in Figure 2. Detailed results of the uni- and multivariable Cox regression analyses are reported in Table 2.

**Table 2:** Predictors of atrial fibrillation recurrence in the overall patient population.

	**Univariable analysis**			**Multivariable analysis**		
	**HR**	**95%CI**	***p***	**HR**	**95%CI**	***p***
Age	1.01	0.99–1.02	0.444	1.01	0.97–1.02	0.800
Female	1.45	1.04–2.01	**0.028**	1.42	0.94–2.15	0.097
BMI	1.02	0.98–1.06	0.310	1.01	0.97–1.06	0.522
Hypertension	0.98	0.68–1.40	0.910	0.97	0.61–1.35	0.900
Hyperlipidemia	0.86	0.58–1.28	0.465	0.83	0.53–1.31	0.429
Diabetes	0.73	0.43–1.23	0.234	0.90	0.51–1.60	0.722
Stroke/TIA	1.07	0.63–1.83	0.792	0.69	0.32–1.49	0.347
Obstructive CAD	1.11	0.65–1.90	0.698	1.86	0.98–3.52	0.057
Thyroid gland disease	1.09	0.66–1.78	0.741	1.01	0.55–1.84	0.987
eGFR <60 ml/min/1.73 m^2^	1.38	0.94–1.51	0.097	1.37	0.88–2.13	0.163
LVEF <50%	1.67	0.96–2.90	0.069	1.40	0.68–2.89	0.357
iLAV	1.01	1.00–1.02	0.149	1.00	0.99–1.02	0.871
E/A ratio	0.80	0.50–1.28	0.352	0.80	0.48–1.34	0.394
LAAV	1.04	0.99–1.10	0.103	1.02	0.95–1.09	0.622
LAA flow velocity	1.01	1.00–1.02	**0.022**	1.01	1.00–1.02	0.087
LAA orifice area	1.00	1.00–1.00	**0.028**	1.00	1.00–1.00	0.327
LAA morphology						
Cauliflower	Ref	Ref	…	Ref	Ref	…
Windsock	1.13	0.78–1.65	0.511	1.35	0.88–2.08	0.166
Chicken wing	1.22	0.73–2.05	0.447	1.51	0.81–2.82	0.197
Swan	1.46	0.73–2.92	0.286	1.73	0.79–3.76	0.168
**LAA abutting LSPV**	**1.53**	**1.09**–**2.14**	**0.013**	**1.55**	**1.04**–**2.31**	**0.030**

*AF, atrial fibrillation; BMI, body mass index; CAD, coronary artery disease; eGFR, estimated glomerular filtration rate; iLAV, body surface area-indexed left atrial volume; LA, left atrium; LAA, left atrial appendage; LAAV, left atrial appendage volume; LVEF, left ventricular ejection fraction; LSPV, left superior pulmonary vein; TIA, transient ischemic attack. The bold variables were statistically significant*.

Subgroup analysis was performed in patients who were ablated with contact force-sensing ablation catheter (*n* = 220) to determine predictors of AF recurrence. Abutting LAA-LSPV was associated with a higher rate of AF recurrence in the univariable analysis (HR = 1.68; 95%CI = 1.05–2.66; *p* = 0.029). However, based on the multivariable analysis, it was not an independent predictor of AF-free survival in this subgroup of patients (adjusted HR = 1.73; 95%CI = 0.95–3.17; *p* = 0.075). Detailed results of the subgroup analysis are shown in Table 3.

**Table 3:** Predictors of atrial fibrillation recurrence in patients who were ablated with contact force sensing catheter.

	**Univariable analysis**			**Multivariable analysis**		
	**HR**	**95%CI**	***p***	**HR**	**95%CI**	***p***
Age	1.01	0.99–1.03	0.433	1.01	0.97–1.04	0.604
Female	1.36	0.87–2.13	0.177	1.06	0.60–1.90	0.838
BMI	1.03	0.98–1.08	0.221	1.03	0.97–1.09	0.404
Hypertension	0.80	0.50–1.28	0.357	0.71	0.45–1.33	0.465
Hyperlipidemia	0.97	0.59–1.62	0.916	1.08	0.58–2.01	0.809
Diabetes	1.35	0.69–1.62	0.377	2.13	0.57–2.01	0.060
Stroke/TIA	0.48	0.17–1.31	0.151	0.54	0.16–1.86	0.329
Obstructive CAD	0.95	0.45–1.97	0.882	1.48	0.58–3.76	0.407
Thyroid gland disease	1.36	0.75–2.47	0.311	1.10	0.50–2.44	0.813
eGFR <60 ml/min/1.73 m^2^	1.01	0.61–1.70	0.973	1.23	0.63–2.40	0.541
LVEF <50%	1.58	0.76–3.30	0.219	1.63	0.58–4.58	0.356
iLAV	1.00	0.98–1.01	0.749	0.99	0.97–1.01	0.456
E/A ratio	0.57	0.27–1.17	0.125	0.58	0.56–1.31	0.194
LAAV	1.03	0.96–1.10	0.442	1.03	0.93–1.14	0.632
LAA flow velocity	1.01	1.00–1.02	0.151	1.01	0.99–1.02	0.264
LAA orifice area	1.00	1.00–1.00	0.460	1.00	1.00–1.00	0.198
LAA morphology						
Cauliflower	Ref	Ref	…	Ref	Ref	…
Windsock	1.35	0.81–2.25	0.246	1.28	0.68–2.41	0.446
Chicken wing	1.28	0.64–2.57	0.483	1.45	0.59–3.57	0.419
Swan	1.29	0.51–3.27	0.593	1.15	0.37–3.56	0.807
LAA abutting LSPV	1.68	1.05–2.66	**0.029**	1.73	0.95–3.17	0.075

*AF, atrial fibrillation; BMI, body mass index; CAD, coronary artery disease; eGFR, estimated glomerular filtration rate; iLAV, body surface area-indexed left atrial volume; LA, left atrium; LAA, left atrial appendage; LAAV, left atrial appendage volume; LVEF, left ventricular ejection fraction; LSPV, left superior pulmonary vein; TIA, transient ischemic attack. The bold variables were statistically significant*.

## Discussion

### Main Finding

Recurrence of AF is generally considered to be related to electrical reconnection between LA and PVs. However, other, less well-understood mechanisms may also be involved. To our knowledge, this is the first study demonstrating that an abutting LAA-LSPV predisposes patients to a higher risk of AF recurrence after catheter ablation for paroxysmal AF. Subgroup analysis of patients who were ablated with contact force-sensing catheter also showed a higher risk of AF recurrence in case of abutting LAA-LSPV.

### Role of LAA-LSPV Spatial Relationship in AF Recurrence

The optimal endpoint of PVI procedures is the electrical isolation of all PVs. This needs to be confirmed by multipolar catheters inserted into the PVs (entrance block) and the inability to capture the LA from the PVs (exit block). The assessment of the bidirectional block is not always straightforward. Far-field capture of LAA may occur during pacing from the LSPV despite the presence of entrance block in up to 16% of cases (pseudo-pulmonary vein exit conduction), but the misinterpretation caused by this phenomenon can be avoided by reducing the pacing output to lose far-field LAA capture but sustain the local PV capture (14, 15). The precise verification of PVI can be strengthened by pacing the LAA as it makes the differentiation between local PV potential and LAA far-field signal possible. In the current study, we performed LAA pacing after PVI in all cases to eliminate the electrical signals' misinterpretation.

The junctions of PVs and LA are highly complex structures with marked heterogeneity of tissue types around the PV ostia. Fibers of the atrial myocardium extend into and around the PVs; moreover, longer sleeves are also frequent (5–10 mm on average), especially in the superior PVs (16). The main branch of the LSPV courses in the anterior direction; thus, this vein is apposed for variable length with the posterior wall of the proximal LAA. Prior studies have demonstrated that conduits may be present between the non-ostial segment of LSPV and the posterior aspect of LAA (16–19). The ligament of Marshall might serve as an electrically active epicardial conduit bypassing the LA–PV junction (17, 18). In such cases, ostial ablation alone may not be sufficient to achieve total PV isolation, but additional ablations may be considered, e.g., ablation of the ligament of Marshall (18).

Regional differences in LA wall thickness are well-known. LA ridge is a structure located between the left-sided PVs ostia and the orifice of the LAA, its wall being thicker as compared to other regions surrounding the PVs (20, 21). A previous study evaluated anatomical properties of the LA ridge in 200 dissected human hearts. “Ridge” was defined as a prominent fold of tissue between left PVs and LAA. At the same time, cases, where the transition between the two structures was not that noticeable were considered as “ridge absent.” Based on their results, the ridge was observed in 59.5% of specimens, and it was absent in the remaining 40.5% of cases. The occurrence of a prominent fold is likely when the LSPV and LAA are in a close anatomical relationship. In such cases, the ridge might be even thicker, which could result in a worse AF-free survival after ablation (22). Another previous study found that bilateral ridge ablation improves arrhythmia-free survival compared to PVI-only in case of paroxysmal AF (23).

The importance of the current findings arises from the high percentage of affected patients, as 54% of our studied population had an abutting LAA-LSPV. We would like to highlight that the evaluation of the abutting vs. non-abutting LAA-LSPV on the CT scan is very simple, quick, and reproducible. Our results indicate that LAA might play a very significant role in the mechanism of paroxysmal AF in those cases when LSPV and LAA are closely juxtaposed. Abutting LAA-LSPV proved to be the most prominent independent predictor of AF recurrence in our study population. The most likely cause of a higher recurrence rate in these cases is the thicker wall of the LA ridge compared to non-abutting cases, but electrical connections between LSPV and LAA might also play a role. Previously reported predictors of AF recurrence are non-modifiable (e.g., age, female gender, LA enlargement). Our data suggest the need for further trials to examine the potential benefit of additional ablation beyond the PVs (e.g., bilateral ridge ablation or ablation of the ligament of Marshall) in case of patients with paroxysmal AF and abutting LAA-LSPV.

A pre-procedural CT scan can be helpful in procedural planning. It is well-known that certain PV anatomical variations might influence the ablation's outcome. Moreover, our current study showed that the anatomical relationship of the LAA and LSPV also impacts the PVI's efficacy. Thus, the pre-procedural CT might enhance an individual risk-benefit analysis and could help to optimize patient selection before catheter ablation.

### Study Limitations

Although this was a single-center retrospective study, a large number of patients were included. Entrance and exit block were confirmed by LAA pacing and atrial non-capture during ostial PV pacing, but the potential ability to capture the LAA from non-ostial LSPV segments was not investigated. Furthermore, ablations with contact force sensing catheters were guided by the currently available parameter, which was force-time integral at that time. Thus, the presented data should be evaluated with recently developed ablation techniques (e.g., high power ablation, CLOSE-protocol). ECG-documented recurrence is still the most widely used definition for the evaluation of AF ablation's efficacy. Although unscheduled visits were scheduled in case of arrhythmia symptoms, the patients did not receive transtelephonic ECG device. Thus, there is a possibility that some short AF episodes were missed during the follow-up, resulting in a lower recurrence rate.

## Conclusion

Our study showed that abutting LAA-LSPV predisposes patients for AF recurrence after pulmonary vein isolation for paroxysmal AF.

## Data Availability Statement

The raw data supporting the conclusions of this article will be made available by the authors, without undue reservation.

## Ethics Statement

The studies involving human participants were reviewed and approved by Institutional Ethics Committee, Semmelweis University. The patients/participants provided their written informed consent to participate in this study.

## Author Contributions

NS and JS: collection of data, concept and design, statistical analysis, and draft preparation. BS, ZS, SH, LS, and GO: collection and interpretation of data and draft revision. MK, GS, KN, MM, JS, VD, JB, PM-H, BM, and LG: interpretation of data and revision of the manuscript. All authors provided approval for publication of the content, and agree to be accountable for all aspects of the work in ensuring that questions related to the accuracy or integrity of any part of the work are appropriately investigated and resolved.

## Funding

This research was supported by the National Research, Development and Innovation Office of Hungary (NKFIA; NVKP_16-1-2016-0017 National Heart Program). The research was financed by the Thematic Excellence Programme (Tématerületi Kiválósági Program, 2020-4.1.1.-TKP2020) of the Ministry for Inn ovation and Technology in Hungary, within the framework of the Therapeutic Development and Bioimaging programmes of the Semmelweis University.

## Conflict of Interest

The authors declare that the research was conducted in the absence of any commercial or financial relationships that could be construed as a potential conflict of interest.

## Publisher's Note

All claims expressed in this article are solely those of the authors and do not necessarily represent those of their affiliated organizations, or those of the publisher, the editors and the reviewers. Any product that may be evaluated in this article, or claim that may be made by its manufacturer, is not guaranteed or endorsed by the publisher.
