# Supplementary material for: Abutting Left Atrial Appendage and Left Superior Pulmonary Vein Predicts Recurrence of Atrial Fibrillation After Point-by-Point Pulmonary Vein Isolation
Source: Front Cardiovasc Med. 2022 Feb 15;9:708298. doi: 10.3389/fcvm.2022.708298 (PMC8885731; doi:10.3389/fcvm.2022.708298)
Supplement: Supplementary file 1 [file Table_1.DOCX]

**Flowchart of patient enrollment.**

| Patients who underwent cardiac CTA before PVI between 2014 and 2017 (n=1280) | |
| --- | --- |
| Non-paroxysmal AF | n=458 |
| n=822 | |
| Not initial AF ablation | n=322 |
| n=500 | |
| Non-diagnostic cardiac CTA image quality | n=47 |
| n=453 | |
| Not radiofrequency point-by-point ablation | n=25 |
| n=428 | |
